# Fluorogenic labeling and single-base resolution analysis of 5-formylcytosine in DNA†
†Electronic supplementary information (ESI) available. See DOI: 10.1039/c7sc03685j



**DOI:** 10.1039/c7sc03685j

**Published:** 2017-09-04

**Authors:** Chaoxing Liu, Yafen Wang, Wei Yang, Fan Wu, Weiwu Zeng, Zonggui Chen, Jinguo Huang, Guangrong Zou, Xiong Zhang, Shaoru Wang, Xiaocheng Weng, Zhiguo Wu, Yu Zhou, Xiang Zhou

**Affiliations:** a College of Chemistry and Molecular Sciences , Key Laboratory of Biomedical Polymers of Ministry of Education , The Institute for Advanced Studies , Hubei Province Key Laboratory of Allergy and Immunology , Wuhan University , Wuhan , Hubei 430072 , P. R. China . Email: xzhou@whu.edu.cn ; Fax: +86-27-68756663 ; Tel: +86-27-68756663; b College of Life Science , Wuhan University , Wuhan , Hubei 430072 , P. R. China

## Abstract

5-Formylcytosine (5fC), which plays an important role in epigenetic functions, has received widespread attention in many related fields. Here, we demonstrate a new design for both the fluorogenic switch-on detection and single-base resolution analysis of 5fC through selectively reacting a reagent with 5fC to yield an intramolecular cyclization nucleobase. The generated product, bearing a similar benzothiazole-iminocoumarin scaffold, is highly fluorescent and enables us to qualitatively and quantitatively detect 5fC moieties in γ-irradiated calf thymus DNA. Additionally, losing the exocyclic 4-amino group in 5fC causes the incorporation of dATP through base pairing with the generated nucleobase during polymerase extension, which helped us to analyze the 5fC sites in both single- and double-stranded oligonucleotides. Our Sanger and Illumina sequencing results show great potential in single-base resolution analysis of 5fC. It is hopeful that a similar design may be used for more detection targets.

## Abstract 2

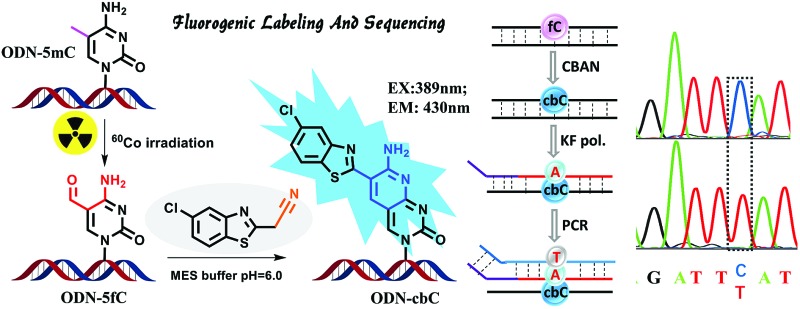
Simultaneous fluorogenic switch-on detection and single-base resolution analysis of 5fC through yielding an intramolecular cyclization nucleobase has been presented.

## Introduction

5-Formylcytosine (5fC) is present at a level of 0.02% to 0.002% that of cytosine in mouse embryonic stem cells (mESCs).^1,2^ It also can be found in many cells and tissues,^3^ such as Hela cells,^4^ HEK293T cells,^5^ hepatocellular carcinoma tissues^6^ and colorectal carcinoma tissues.^7^ It is a transient intermediate in the demethylation of 5-methylcytosine (5mC) by ten-eleven translocation (TET) family enzymes,^8,9^ and it is stable in various genomes,^10^ with mounting evidence to suggest that it plays a vital role in epigenetic functions.^11^ It is associated with gene regulation,^12^ alterations in DNA structures,^13,14^ cell differentiation^15,16^ and some illnesses.^17^ Research on 5fC has accelerated the complete understanding of genetic and epigenetic regulation. 5fC can be effectively fluorogenically labeled by hydrazine,^18^ amine,^19^ amidoxyl^20^ and indole derivatives.^21^ However, 5-formyluracil (5fU), the modified thymine (T) counterpart of 5fC, is more active,^22–24^ which causes many fluorescent reagents to prefer to react with 5fU.^25–27^ As far as we know, there is no report of reagents that can fluorogenically switch on 5fC but that do not disturb the fluorescence detection of 5fU and abasic sites (AP). The difference between 5fC and 5fU is minor, but the reactivity of the aldehyde present in 5fU is more chemoselective, making it a big challenge to design a reagent that can only fluorogenically switch on 5fC.

As for the sequencing of 5fC, the recent discovery of cytosine (C) modifications in genomic DNA has attracted widespread attention and has energized the field of epigenetics.^28^ Balasubramanian and co-workers made a breakthrough in the genome-wide map of 5fC in mESCs using the commercial probe O-(biotinylcarbazoylmethyl)hydroxylamine.^29^ After that, they created a reduced bisulfite sequencing method (redBS-seq) based on the selective chemical reduction of 5fC to 5-hydroxymethylcytosine (5hmC) followed by bisulfite treatment which can detect 5fC in DNA at single-base resolution.^30^ He and co-workers presented a pair of methods, fCAB-seq and fC-Seal, which employ 5fC-selective chemical manipulation to enable base-resolution analysis and its affinity enrichment.^31^ Their studies were noteworthy, systematic and timely. Yi *et al.* creatively reported the fC-CET method (bisulfite-free, selective chemical labeling of 5fC and subsequent C-to-T transition during PCR) for the single-base analysis of 5fC that avoided the DNA degradation of many bisulfite-based methods.^32^ Subsequently, the modified method (CLEVER-seq) was used in single cell sequencing of 5fC.^33^ It is a remarkable and significant finding that reagents undergoing base modification to result in a C-to-T transition during PCR could have wider applications in epigenome sequencing.^32–34^ However, reported reagents have been sparse. Thus, the synthesis of reagents that could be used in bisulfite-free, single-base analysis of 5fC is highly needed.

**Fig. 1:**
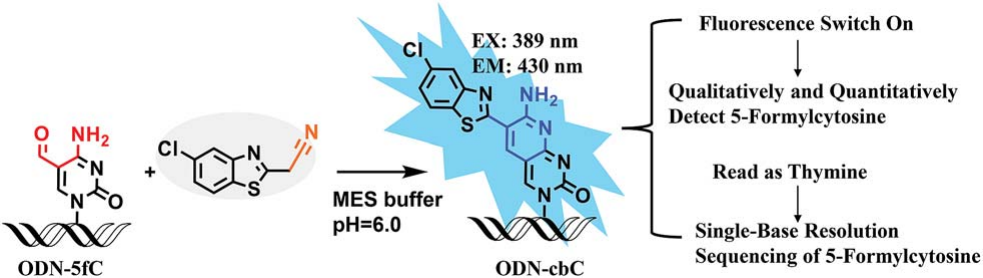
Strategies of both fluorogenic labeling and single-base resolution analysis of 5fC in DNA.

Herein, we provide new insight into meeting the demands of both fluorogenic labeling and single-base resolution analysis of 5fC in DNA. In Fig. 1, 2-(5-chlorobenzo[*d*]thiazol-2-yl)acetonitrile (CBAN) was chosen to selectively react with 5fC to generate a 5-formyl-2′-deoxycytidine-CBAN adduct (CB-C), which is similar to the synthesis that uses 2-benzothiazoleacetonitrile and 2-aminobenzaldehyde to form 2-amino-3-(1,3-benzothiazol-2-yl)-quinoline.^35^ The direct condensation of aminobenzaldehydes and cyano reagents is very effective and highly selective. Other syntheses that do not involve an aminobenzaldehyde structure cannot proceed through intramolecular cyclization with cyano reagents (Scheme S1†).^33,35,36^ Among naturally modified nucleobases, only 5fC contains the same structure. Furthermore, the benzothiazole-iminocoumarin fluorophore possesses excellent photophysical properties, such as a high quantum yield and good photostability.^37–40^ The generated nucleoside CB-C contains a similar scaffold that may be highly fluorescent. Meanwhile, it loses its exocyclic 4-amino group, which is a competent proton donor in 5fC, thus failing to base pair with guanine (G). This leads to C-to-T transition during polymerase extension and base pairing with adenine (A), which can further lead to C–T conversion signals after PCR amplification and sequencing.^32,33^ Thus, we hypothesize that this design can be used in both highly selective fluorescence switch-on detection and single-base resolution analysis of 5fC.

## Results and discussion

### Quantitative and qualitative detection of 5fC

**Fig. 2:**
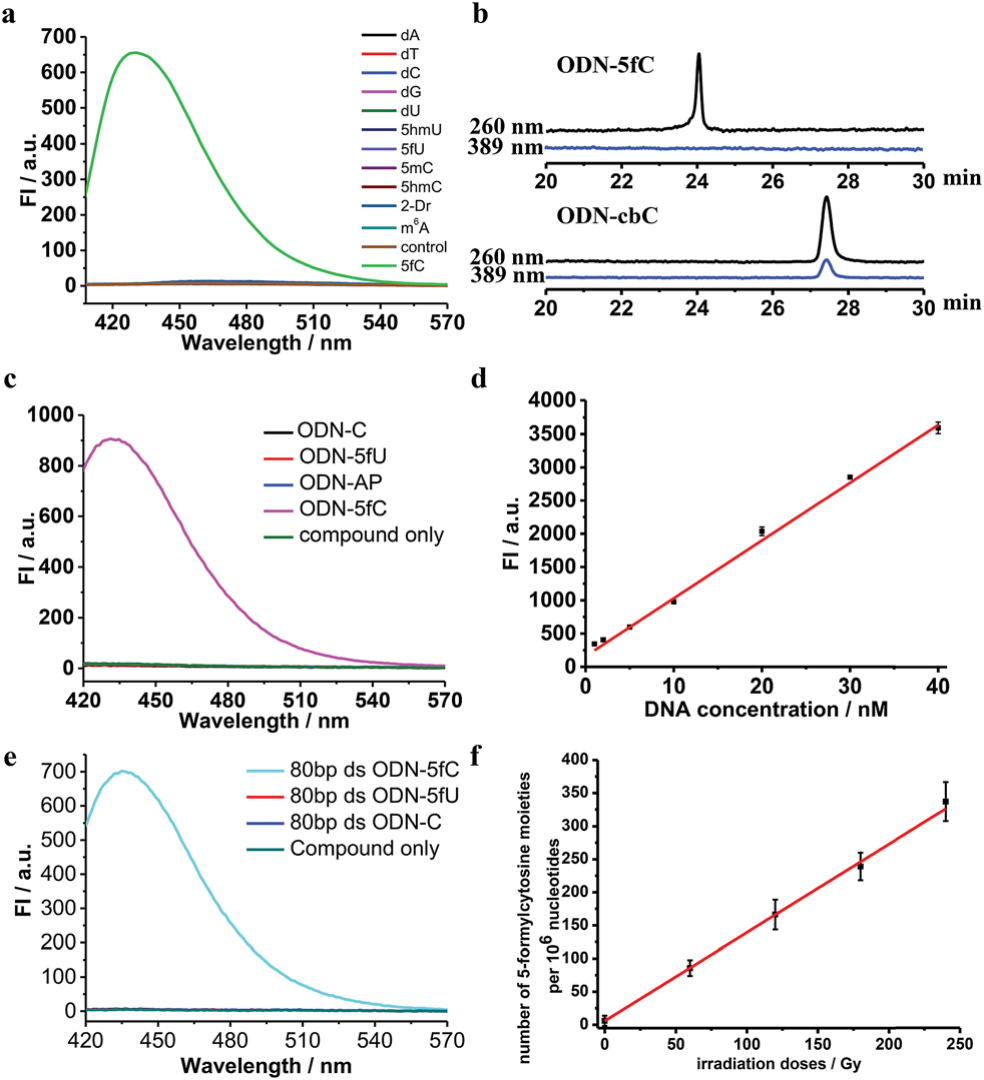
Quantitative and qualitative detection of 5fC. (a) Fluorescence emission spectra (*λ*_ex_: 389 nm, *λ*_em_: 430 nm) of different nucleosides (100 nM) after incubation with CBAN (100 nM) in MES buffer (100 mM, pH 6.0). (b) RP-HPLC trace at *λ* = 260 nm (black) and 389 nm (blue) of ODN-5fC and ODN-cbC, which were labeled by a reaction with CBAN under optimized conditions. (c) Fluorescence emission spectra (*λ*_ex_: 389 nm, *λ*_em_: 430 nm) of ODNs after reaction with CBAN. (d) Correlation of the fluorescence intensity (at 430 nm) of ODN-5fC after fluorogenic labeling by CBAN with DNA concentration. (e) Fluorescence emission spectra (*λ*_ex_: 389 nm, *λ*_em_: 430 nm) of various dsDNAs that contain 5fU, 5fC or only canonical nucleobases after treatment with CBAN. (f) Quantification of 5fC in γ-irradiated calf thymus DNA (50 μg) at different irradiation doses (0–240 Gy) (*λ*_ex_: 389 nm, *λ*_em_: 430 nm).

Firstly, to verify the feasibility of our design, 5-formyl-2′-deoxycytidine was reacted with CBAN in a methanol piperidine solution to cyclize *via* the addition of an exocyclic 4-amino group to the cyano group to give the corresponding nucleotide derivative CB-C (Fig. S12 and S13†). The absorbance and fluorescence emission properties of CB-C were acquired in various buffer solutions. The absorbance was detected at 389 nm, and the fluorescence emission maximum was 430 nm (Fig. S8†). Next, to demonstrate the highly efficient fluorescence-based switch-on detection of 5fC, we treated CBAN with canonical deoxynucleosides and their modifications. Only a dramatic fluorescence enhancement (*λ*_ex_: 389 nm, *λ*_em_: 430 nm) for 5-formyl-2′-deoxycytidine can be observed compared to other deoxynucleosides, such as those containing the 5fU moiety, through direct fluorescence readout without further purification (Fig. 2a). We also reacted 5-formyl-2′-deoxyuridine with CBAN to generate a 5-formyl-2′-deoxyuridine-CBAN adduct (CB-U) without cyclization (Fig. S14†). The following studies revealed that CB-U is not fluorescent and does not affect the qualitative and quantitative fluorescence detection of 5fC. This may be because the decyclization scaffold contributes to the lack of fluorescence.^39,40^ Then, ODN-5fC containing one 5fC site was reacted with CBAN in MES buffer (pH 6.0) at 60 °C for 10 h. Complete conversion to the new product ODN-cbC was recorded using RP-HPLC (monitored at 260 nm and 389 nm) (Fig. 2b). The integrity of the labeled DNAs was confirmed by MALDI-TOF MS (Fig. S1†). The enzymatically digested mononucleosides were analyzed through LC-MS to ensure the reaction of 5fC to yield CB-C (Fig. S4†). In control experiments, we also incubated other ODNs (the 5fC site was replaced by C, 5mC, 5hmC, 5hmU, 5fU and AP) with CBAN under the same conditions. Only 5fU could be labeled with CBAN in the same manner as 5fC, and the other ODNs showed no reactions according to the results of the RP-HPLC and denaturing polyacrylamide gel electrophoresis (PAGE) analyses (Fig. S2 and S3†). Furthermore, the fluorescence intensities of different single-stranded (ss) ODNs after incubation with CBAN under the same conditions demonstrated that CBAN is an excellent reagent for the highly selective fluorogenic tagging of 5fC in DNA, and there was negligible fluorescence observed for the control ODNs, indicating that AP, which also contains aldehydes, did not perturb the fluorescence detection of 5fC (Fig. 2c). A linear correlation between the concentration of ODN-5fC and fluorescence intensity ranging from 0 nM to 40 nM was also observed (Fig. 2d). All of these results indicate that this is a highly selective and fluorescence-based switch-on method for the qualitative and quantitative detection of 5fC. Since most biological samples bearing 5fC are in double-stranded (ds) forms, it is very important to determine whether CBAN can effectively label 5fC in dsDNAs. Therefore, we used a series of 80-bp dsDNAs (containing 5fC or 5fU sites or just canonical nucleosides) as a model test. The fluorescence intensities proved that the reagent can also fluorogenically label 5fC in dsDNA compared to that of other controls, hinting at more possibilities for CBAN in future applications (Fig. 2e).

### Quantification of 5fC in γ-irradiated calf thymus DNA at different irradiation doses

Recently, Wagner *et al.* reported that 5fC can be generated from the oxidation of 5mC by being exposed to ionizing radiation in an oxygenated aqueous solution.^41^ With all of these encouraging findings, we commenced applying our method to complex biological samples (γ-irradiated calf thymus DNA) to qualitatively and quantitatively detect 5fC formation. First, 5-methyl-2′-deoxycytidine and ODN-5mC (pre-dissolved in an oxygenated aqueous solution) were subjected to ^60^Co irradiation (17.4 Gy min^–1^, 60 min) at room temperature. After that, the LC-MS data showed the exact generation of 5fC in both γ-irradiated 5-methyl-2′-deoxycytidine and enzymatically digested γ-irradiated ODN-5mC solutions (Fig. S5 and S6†). Next, we treated CBAN with 5-methyl-2′-deoxycytidine, γ-irradiated 5-methyl-2′-deoxycytidine, ODN-5mC, γ-irradiated ODN-5mC, ODN-C and γ-irradiated ODN-C solutions. We found that only solutions containing a γ-irradiated 5mC moiety were highly fluorescent (Fig. S9†). Then, we prepared γ-irradiated calf thymus DNA (0.5 mg mL^–1^, prior to being bubbled with oxygen for 1 h) at various doses of ^60^Co irradiation (0–240 Gy at 1.84 Gy min^–1^). After incubation with CBAN, the fluorescence spectra were measured using excitation at 389 nm and emission at 430 nm. We obtained 1.30 5fC moieties per 10^6^ nucleotides per Gy (1.84 Gy min^–1^, ^60^Co irradiation) upon evaluation of the slope of the graph shown in Fig. 2f. Comparatively, quantification can also be performed using quantitative mass spectrometry. So we used reported LC-MS/MS analysis methods to detect enzymatically digested γ-irradiated calf thymus DNA^42^ and found 1.27 5fC moieties per 10^6^ nucleotides per Gy (Fig. S7†). Our results are in reasonable agreement with that of Wagner *et al.*, who reported a formation rate of 0.45 fC moieties per 10^6^ nucleotides per Gy (1.2 Gy min^–1^).^41^ The slightly higher result might be due to more dissolved oxygen, a higher dose per minute or different batches of biological samples. It is worth noting that the modified nucleoside was also found to be induced by a hydroxyl radical produced from a Fenton-type reaction.^43^ These reasons all might contribute to a higher content of 5fC in the assays. The quantification of 5fC in γ-irradiated calf thymus DNA at different irradiation doses suggests that this is a highly efficient fluorescence-based switch-on detection method of 5fC in DNA. In addition, quantification using quantitative mass spectrometry verified the feasibility of our method. There is no denying that the changeable levels of natural nucleobase modifications in living cells can be obtained using MS-based isotope tracing,^44^ and our fluorescence-based switch-on detection method lacks such advantages. Even if the directly competing method (MS with isotope standards) bears the properties of warmer conditions, lower detection limit and higher sensitivity,^5,7,45^ our fluorescence-based switch-on detection method does not require expensive MS instruments or professional operation and has many properties such as faster detection, easier operation, visualization technology for qualitative detection and so on which can also be effective supplements in some situations.

### Primer extension assay with KF exo-DNA polymerase

**Fig. 3:**
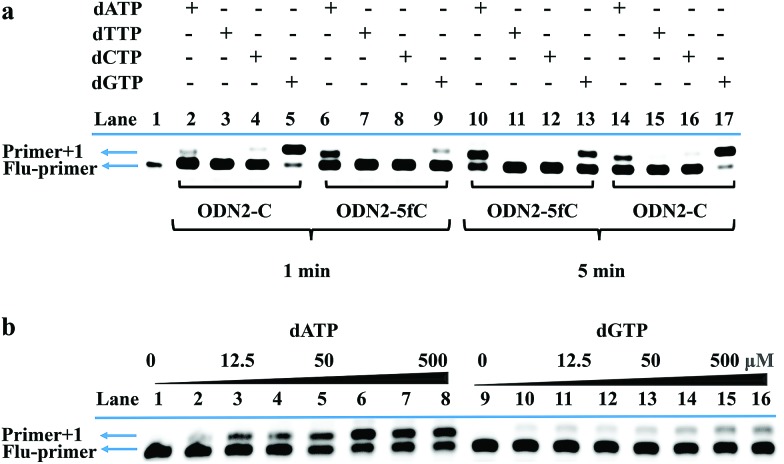
Primer extension assay with KF exo-DNA polymerase. (a) Incorporation of dATP, dTTP, dCTP and dGTP opposite the original 5fC site of ODN2-5fC (after being labeled with CBAN) for 1 and 5 min. ODN2-C in which the 5fC site was replaced by C was used as a control. (b) Incorporation of dATP or dGTP into the corresponding primers of ODN2-5fC (after reaction with CBAN) with various concentrations of dATP or dGTP (0–500 μM).

On the other hand, to determine whether such a cyclic adduct could enable the C-to-T transition of 5fC during PCR amplification, similar primer extension assays were prepared.^32^ ODN2-5fC and ODN2-C (where the 5fC sites were replaced by C) were reacted with CBAN. After primer extension with Klenow fragment exo-(KF exo-) DNA polymerase, the mixture solution was subjected to denaturing PAGE analysis, revealing the incorporation of dATP into the primers of the ODN2-5fC template (after reaction with CBAN) rather than dGTP when compared to ODN2-C (Fig. 3a). To compare the incorporation efficiency of dATP and dGTP, various concentrations of dATP and dGTP were used in the primer extension reaction. The denaturing PAGE analysis demonstrated that dGTP showed a similar increasing tendency to dATP, but with an obviously slower incorporation rate (Fig. 3b). All of these results showed that the cyclization nucleoside generated from 5fC effectively pairs with A, which encouraged us to do more tests.

### Single-base resolution analysis of 5fC

**Fig. 4:**
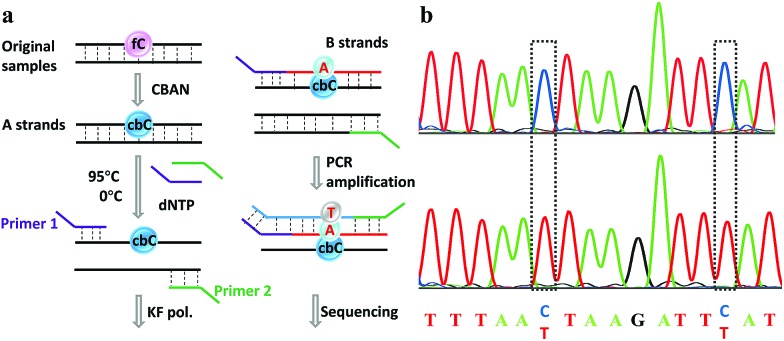
Single-base resolution analysis of 5fC. (a) Illustration of a strategy for samples containing 5fC moieties. (b) Sanger sequencing analysis before and after CBAN treatment of the oligonucleotides containing two sites of 5fC. The original 5fC sites are surrounded by dotted lines.

Finally, an 80-mer oligonucleotide bearing two 5fC sites (80-SS-fC) was labeled with CBAN, PCR amplified and then subjected to Sanger sequencing. The Sanger sequencing results of 80-SS-fC before and after the reaction with CBAN showed exact effective C-to-T signals (Fig. S10†). It is also important to ensure that this reagent can be applied to dsDNAs. The above 80 bp-dsODN-fC was used as a model test. Samples containing 5fC were reacted with CBAN to generate cyclization nucleosides (Fig. 4a, A strands). After the chemicals were removed, excessive primers (primer 1 and 2) and dNTP were added at 95 °C for denaturation for 8 minutes. Then, the samples were immediately put on ice for 5 minutes. Next, KF exo-DNA polymerase and buffer were added at 37 °C for 1 cycle of primer extension to achieve dATP incorporation through pairing with CB-C in the complementary strands (B strands). Then, we obtained a high T signal in the original 5fC sites from the sequencing of the amplification of the B strands. To our delight, the Sanger sequencing results of the 80 bp-dsODN-fC before and after being treated with CBAN revealed an effective C-to-T conversion in the application to the dsDNAs (Fig. 4b). We also investigated multiple 5fC-containing dsDNA segments (four sites) using an Illumina sequencing system. The DNA was incubated with CBAN and then treated using the above protocol. After purification, it was subjected to Illumina library preparation using the ThruPLEX® DNA-seq Kit (Rubicon genomics). An obvious difference in the conversion ratios between the CBAN-treated and untreated samples suggested that 5fC can be detected at base resolution when using the Illumina sequencing method (Fig. S11†). In comparison, peroxotungstate can be used to oxidize 5hmC to thT to undergo base pairing with A and therefore be applied in the analysis of 5hmC at base-resolution.^34^ However, it can’t be directly applied to the dsDNAs besides with treatment under thermal cycle conditions. A 1,3-indandione derivative^32^ and malononitrile^33^ were also employed in the analysis of 5fC at base-resolution. However, the reaction times of the 1,3-indandione derivative (24 h) and malononitrile (20 h) were longer than CBAN (10 h). 2,3,3-trimethylindole derivatives can react with 5fC to produce a fluorescent nucleobase in 3 M NaCl solution.^21^ However, the yield and selectivity of the reagents restricted their applications because it can also fluorogenically switch-on 5fU and the condensation was not completed even when the best optimal labeling conditions for 5fC were chosen. Even though similar reagents were shown before, bearing the similar property of converting the coding potential of 5fC from G to A, the reagent we have presented here is also novel as a bifunctional molecule for the simultaneous selective and fluorogenical switch-on detection and analysis of 5fC in a single-base resolution.

## Conclusions

In conclusion, herein we have described a conceptually new type of reagent for use in fluorogenic switch-on detection and base-resolution analysis of 5fC through a selective reaction with 5fC to generate an intramolecular cyclization nucleobase. As far as we know, this is the first reagent that can fluorogenically switch on 5fC while leaving 5fU and AP undisturbed. Its excellent fluorescence properties enabled us to detect the 5fC moieties of γ-irradiated calf thymus DNA. Additionally, loss of the exocyclic 4-amino group, which is a competent proton donor in 5fC, allows the nucleobase to undergo base pairing with A and leads to C-to-T conversion during polymerase extension. We also revealed the possibility of single-base resolution analysis of 5fC for both single- and double-stranded oligonucleotides by employing this reagent. The Sanger and Illumina sequencing results showed the great potential of this complex in the single-base resolution analysis of 5fC. A similar design could be further applied to both qualitative and quantitative detection and single-base resolution analysis of natural C modifications in more cell and tissue studies in the near future.

## Conflicts of interest

There are no conflicts to declare.
