# Supplementary material for: Fluorogenic labeling and single-base resolution analysis of 5-formylcytosine in DNA
Source: Chem Sci. 2017 Sep 4;8(11):7443–7. doi: 10.1039/c7sc03685j (PMC5674178; doi:10.1039/c7sc03685j)
Supplement: Supplementary file 1 [file SC-008-C7SC03685J-s001.pdf]

Electronic Supplementary Material (ESI) for Chemical Science.
This journal is © The Royal Society of Chemistry 2017

Supporting Information

Fluorogenic Labeling and Single-Base Resolution Analysis of 5-Formylcytosine in DNA

Chaoxing Liu[1, †], Yafen Wang[1, †], Wei Yang[1], Fan Wu[1], Weiwu Zeng[1], Zonggui Chen[2], Jinguo Huang[1], Guangrong Zou[1], Xiong Zhang[1], Shaoru Wang[1], Xiaocheng Weng[1], Zhiguo Wu[2], Yu Zhou[2] and Xiang Zhou[1, *]

[1] College of Chemistry and Molecular Sciences, Key Laboratory of Biomedical Polymers of Ministry of Education, The Institute for Advanced Studies, Hubei Province Key Laboratory of Allergy and Immunology, Wuhan University, Wuhan, Hubei, 430072, P. R. China.

[2] College of Life Science, Wuhan University, Wuhan, Hubei, 430072, P. R. China.

Table of Contents

1	General methods and materials	S1
2	DNA MALDI-TOF Mass Spectra	S2
3	HPLC data	S3
4	Polyacrylamide gel electrophoresis analysis	S5
5	LC-MS about digesting DNAs	S6
6	LC-MS/MS quantification	S11
7	UV absorption spectra and fluorescent emission spectra	S12
8	Sanger sequencing	S13
9	Illumina sequencing	S14
10	Synthesis	S15
11	Table of oligonucleotides sequences	S19
12	Reference	S20

1. General methods and materials

All chemicals were purchased from Adamas-beta® (Shanghai, China) unless stated otherwise. ^{1}H NMR, ^{13}C NMR spectra were collected on Varian Mercury 300 or 400 spectrometers, respectively. HRMS was acquired with Thermo Scientific™ Dionex Ultimate 3000 hybrid LTQ Orbitrap Elite Velos Pro (Thermo Scientific, USA). All the oligonucleotides were synthesized and purified by GeneCreate Co., Ltd. (Wuhan, China) unless stated otherwise. And the oligonucleotides containing 5-formylcytosine (ODN-5fC, ODN2-5fC) were bought from Takara Biotechnology (Dalian, China). The oligonucleotides containing 5-formyluracil (ODN-5fU) were synthesized using a 5-formyluracil phosphoramidite[1]. ODN-AP was synthesized through ODN-U (one T site was replaced by uracil) treating with Uracil DNA Glycosylase (Invitrogen™, USA). DNA concentration was quantified by NanoDrop 2000c (Thermo Scientific, USA). DNA MALDI-TOF Mass Spectra were collected on MALDI-TOF-MS (Shimadzu, Japan). Gel Imaging was collected in Pharos FX Molecular imager (Bio-Rad, USA). The nucleic acid stains Super GelRed (NO.: S-2001) was bought from US Everbright Inc. (Suzhou, China). And the YeaRed Nucleic Acid Gel Stain (NO.: 10202ES76) was purchased from YEASEN Biotechnology Co. Ltd., (Shanghai, China). UV absorption spectra were acquired with SHIMADZU UV-2550. Fluorescence emission spectra were recorded on PerkinElmer LS 55 (PerkinElmer, USA). pH was measured with Mettler Toledo, FE20-Five Easy™ pH (Mettler Toledo, Switzerland). LC-MS data were collected with the Agilent™ 1220 Infinity LC combined with the 6120 Single Quadrupole mass spectrometer (Agilent Technologies). Degradase Plus and enzyme reaction buffer were purchased from Zymo Research (Zymo Research, USA).

ODN reaction protocol. ODNs (100 μM, 1 μL) were added in the final MES buffer (100 mM, pH=6.0) containing 50 mM CBAN at 60°C for 10 h, respectively.

HPLC analysis of ODNs. ODNs were reacted with CBAN through the above protocol, then HPLC data were performed on LC-6AD (Shimadzu, Japan) which equipped with an Inertsil ODS-SP column (5 μm, 250×4.6 mm) (GL Science lnc. Japan) with mobile phase A (CH_3CN) and B (100 mM TEAA buffer, pH=7.0) with flow rate of 1 mL/min at 35°C (A Conc.: 5-5-30% / 0-5-30 min).

Enzymatic digest of ODNs protocol. DNAs were added in the 10×Degradase Plus reaction buffer (2.5 μL) (Zymo Research) containing Degradase Plus (1 μL) in a final volume of 25 μL at 37°C for 2 h for digesting to its corresponding nucleosides. The solution mixture was filtered by an ultrafiltration tube (3 kDa cutoff, Amicon, Millipore) to remove the enzymes.

Primer-extension assay with KF exo- DNA polymerase. ODN2-5fC and ODN2-C (the 5fC site was replaced by C) were reacted with CBAN, respectively. After purification to removed chemicals, 1.93 pmol of them were used as the templates. Templates, fluorescein-labeled primer, Klenow fragment exo- (KF exo-) DNA polymerase and dXTP (dATP, dTTP, dCTP or dGTP, respectively) were added in the polymerase reaction buffer at 37°C for 1 or 5 min. Finally, the mixture was added the same volume deionized formamide and subjected to 20% denaturing PAGE which was carried out in 1xTBE buffer at a constant voltage of 350 V for about 4 h at room temperature. The Pharos FX Molecular imager was operated in the fluorescence mode (λ_{ex}=488 nm) to give the final polyacrylamide gel electrophoresis figures.

2. DNA MALDI-TOF Mass Spectra

a

5’-GACTCAA**5fC**AGCCGTA-3’ ⟶ 5’- GACTCAA**cbC**AGCCGTA-3’

calculated 4762.8, found 4761.8.

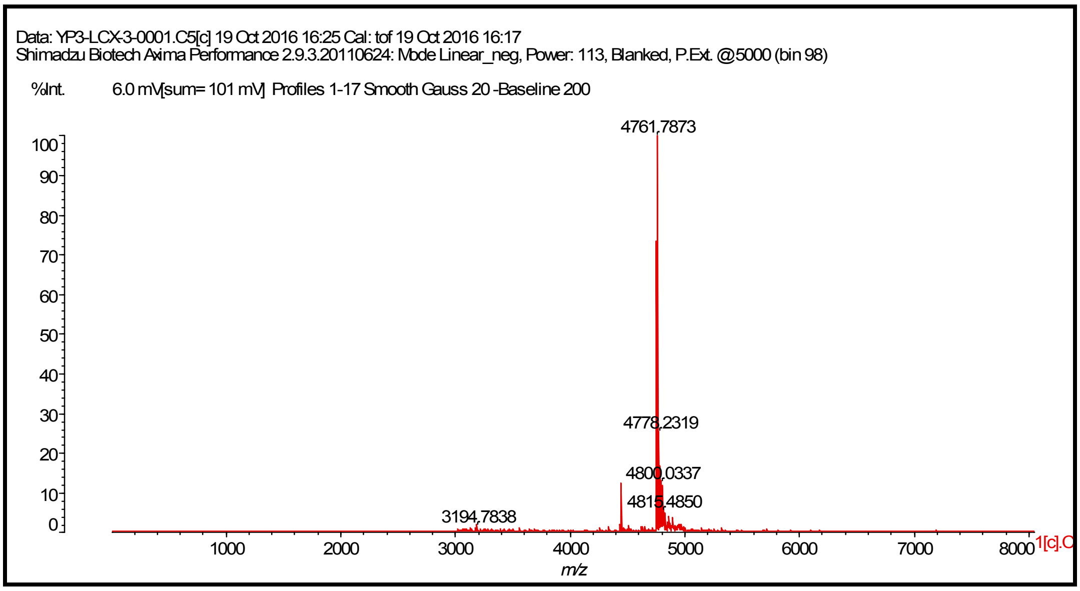

b

5’-AAATCA**5fC**CCTATCCTCCTTCAGGACCAACGTAC-3’ ⟶

5’-AAATCA**cbC**CCTATCCTCCTTCAGGACCAACGTAC-3’

calculated 10160.7, found 10164.4.

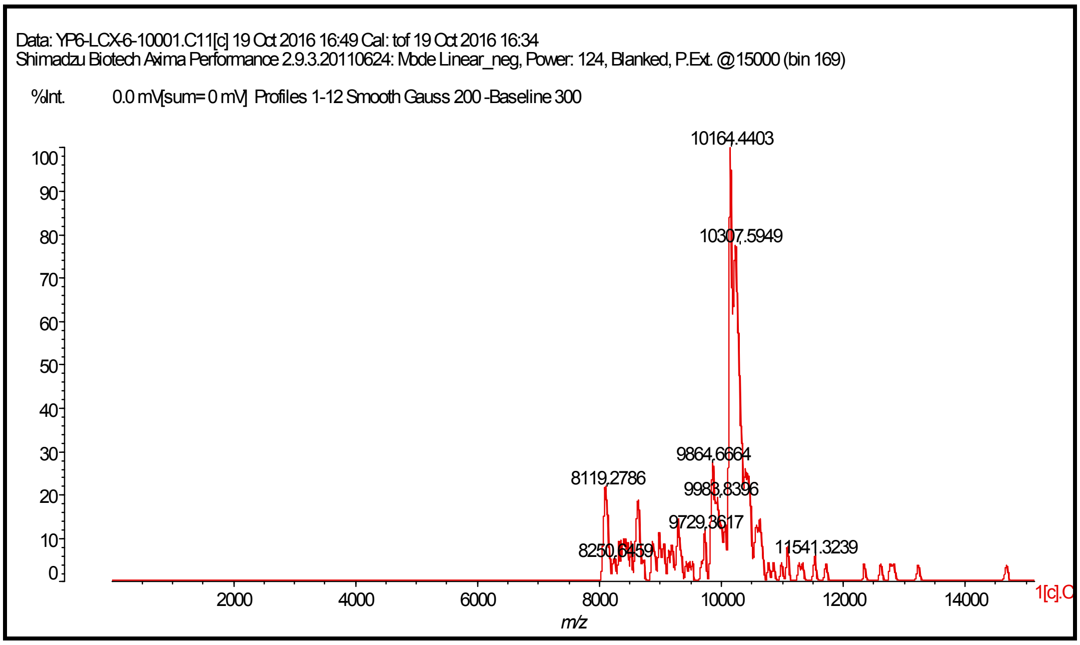

Figure S1. DNA MALDI-TOF Mass Spectra.

(a) MALDI-TOF-spectrum of ODN-cbC; (b) MALDI-TOF-spectrum of ODN2-cbC.

3. HPLC data

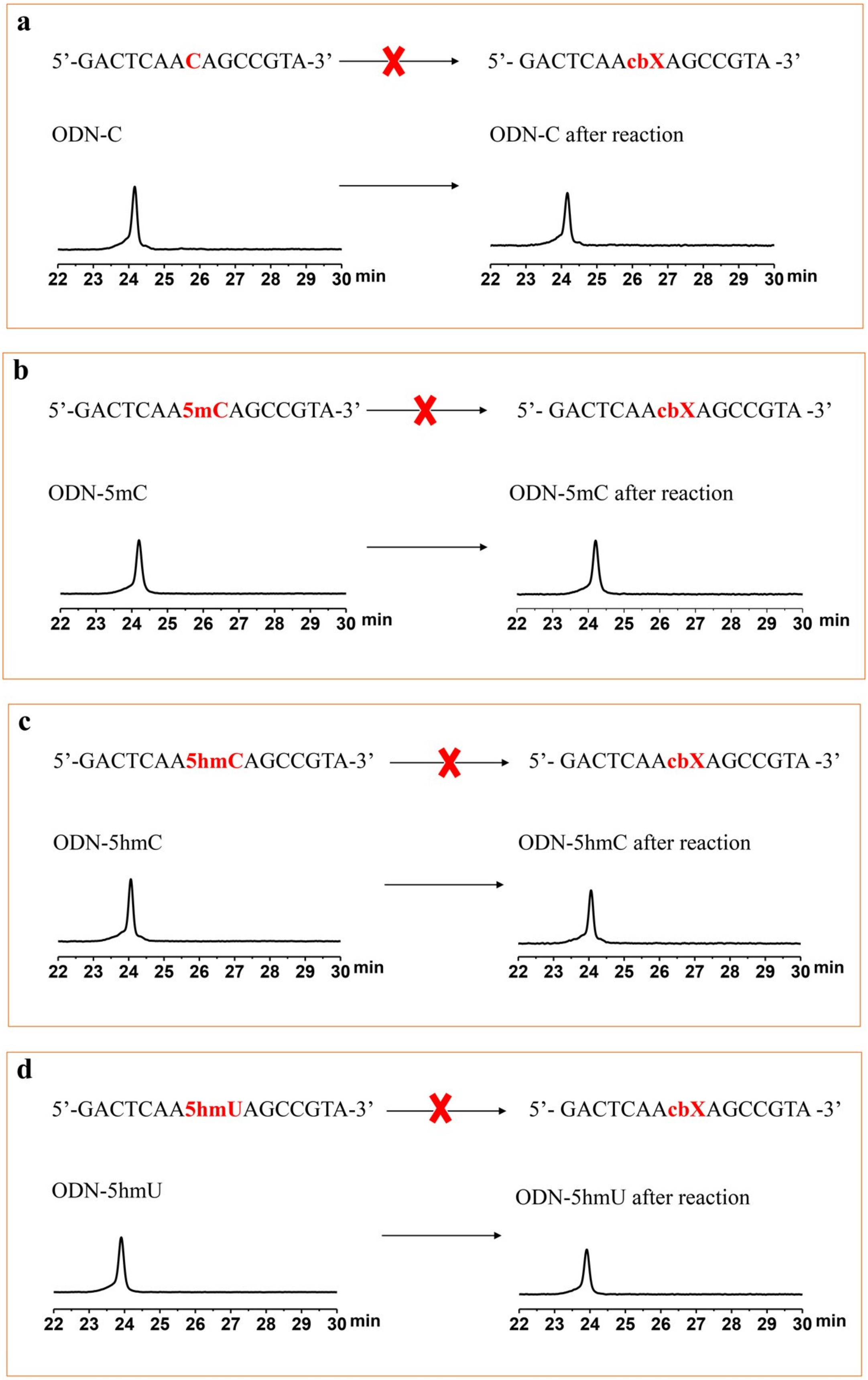
a
5'-GACTCAACAGCCGTA-3'
5'- GACTCAAcbXAGCCGTA -3'
ODN-C
ODN-C after reaction
22 23 24 25 26 27 28 29 30 min
22 23 24 25 26 27 28 29 30 min
b
5'-GACTCAA5mCAGCCGTA-3'
5'- GACTCAAcbXAGCCGTA -3'
ODN-5mC
ODN-5mC after reaction
22 23 24 25 26 27 28 29 30 min
22 23 24 25 26 27 28 29 30 min
c
5'-GACTCAA5hmCAGCCGTA-3'
5'- GACTCAAcbXAGCCGTA -3'
ODN-5hmC
ODN-5hmC after reaction
22 23 24 25 26 27 28 29 30 min
22 23 24 25 26 27 28 29 30 min
d
5'-GACTCAA5hmUAGCCGTA-3'
5'- GACTCAAcbXAGCCGTA -3'
ODN-5hmU
ODN-5hmU after reaction
22 23 24 25 26 27 28 29 30 min
22 23 24 25 26 27 28 29 30 min

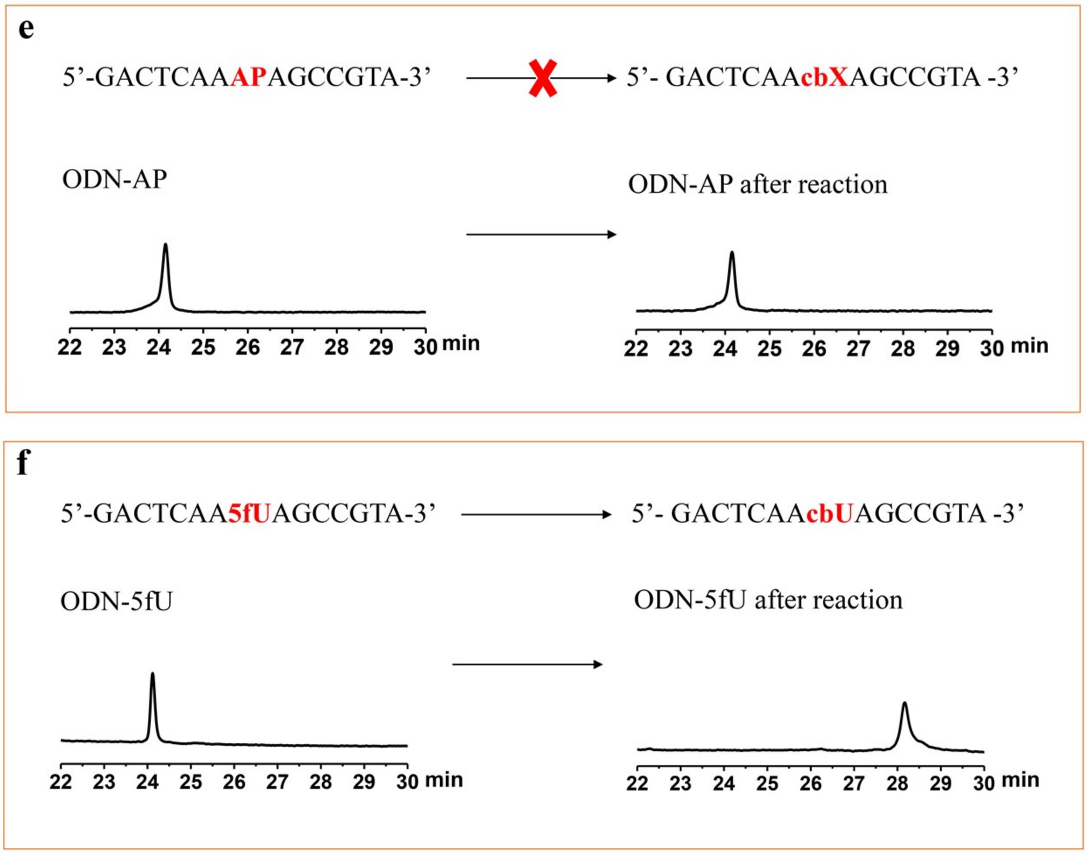

Figure S2. HPLC detection of various ODNs before and after treatment with CBAN. (a) RP-HPLC trace at λ = 260 nm of ODN-C before and after treatment with CBAN. (b) RP-HPLC trace at λ = 260 nm of ODN-5mC before and after treatment with CBAN. (c) RP-HPLC trace at λ = 260 nm of ODN-5hmC before and after treatment with CBAN. (d) RP-HPLC trace at λ = 260 nm of ODN-5hmU before and after treatment with CBAN. (e) RP-HPLC trace at λ = 260 nm of ODN-AP before and after treatment with CBAN. (f) RP-HPLC trace at λ = 260 nm of ODN-5fU before and after treatment with CBAN.

4. Polyacrylamide gel electrophoresis analysis

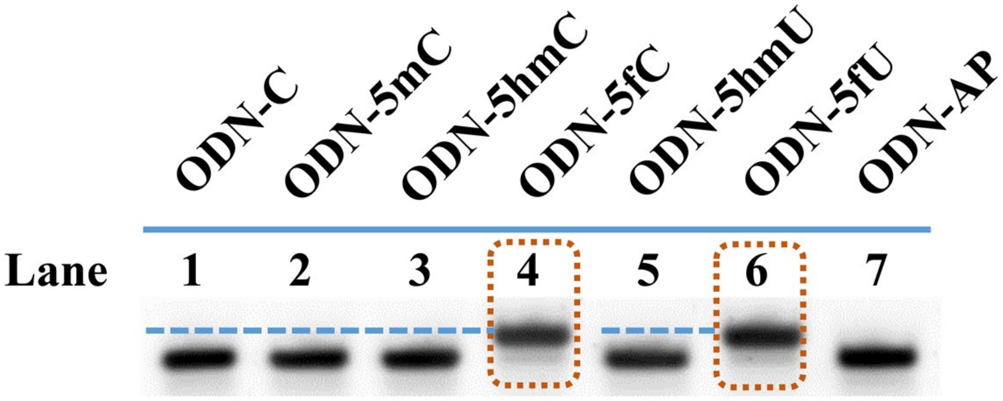

Figure S3. Polyacrylamide gel electrophoresis analysis of various ODNs after treatment with CBAN.

Polyacrylamide gel electrophoresis analysis of ODN-5fC and ODN-5fU after incubation with CBAN (lane 4, 6) (above dash line) after being stained with nucleic acid stains (fluorescence mode, λ_{ex}: 532 nm) in comparison with other control DNAs such as ODN-C (lane 1), ODN-5mC (lane 2), ODN-5hmC (lane 3), ODN-5hmU (lane 5), and ODN-AP (lane 7) under the same conditions (below dash line).

5. LC-MS about digesting DNAs

a

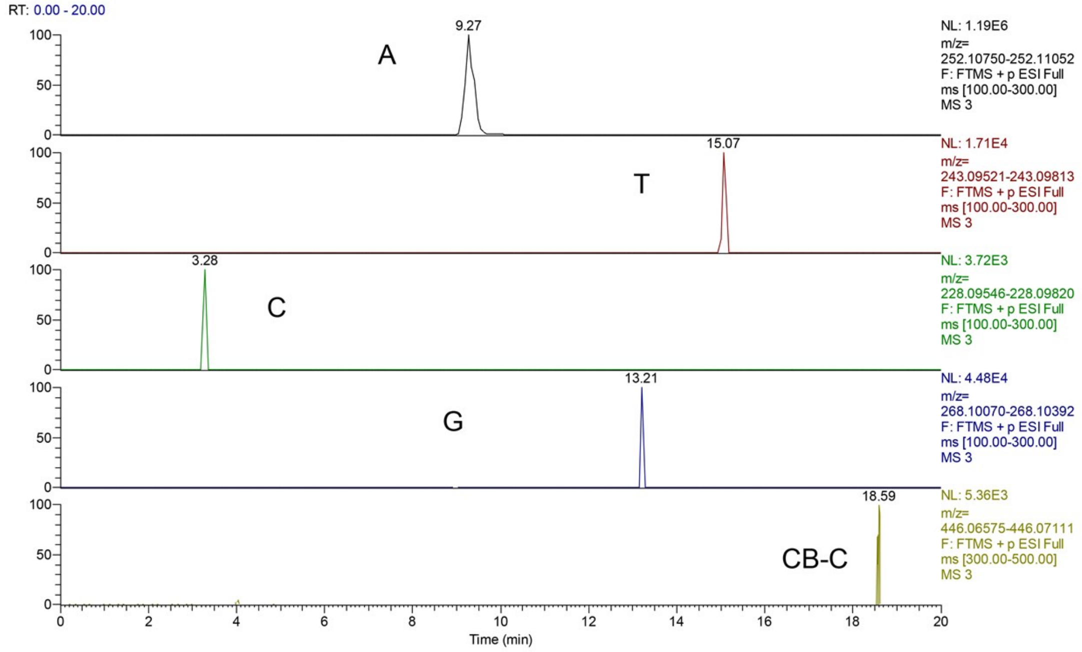

b

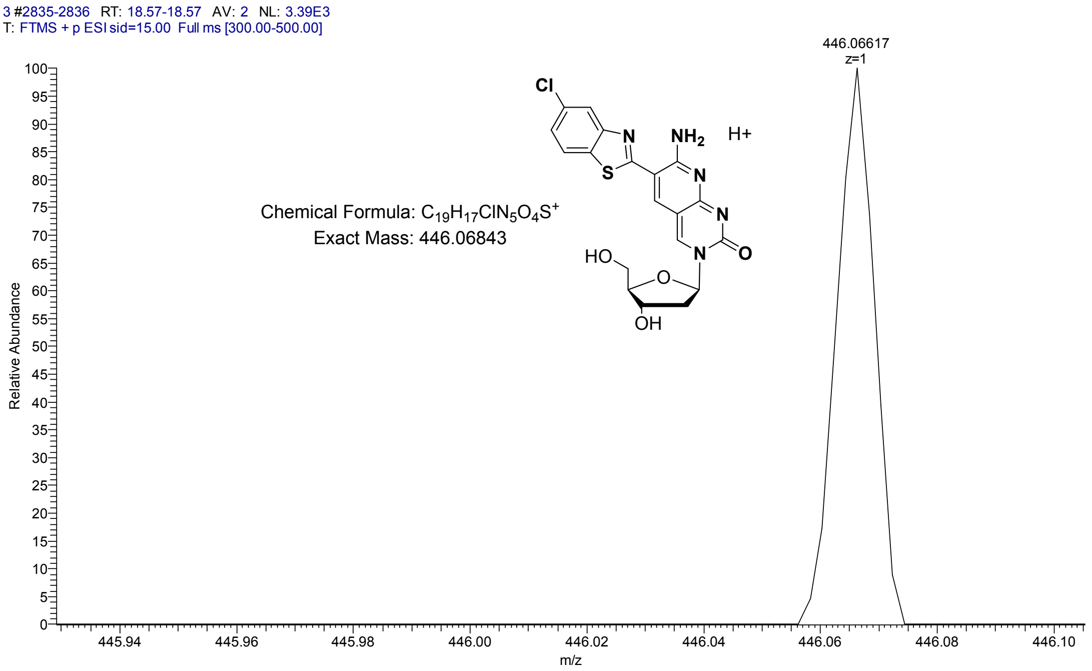

C

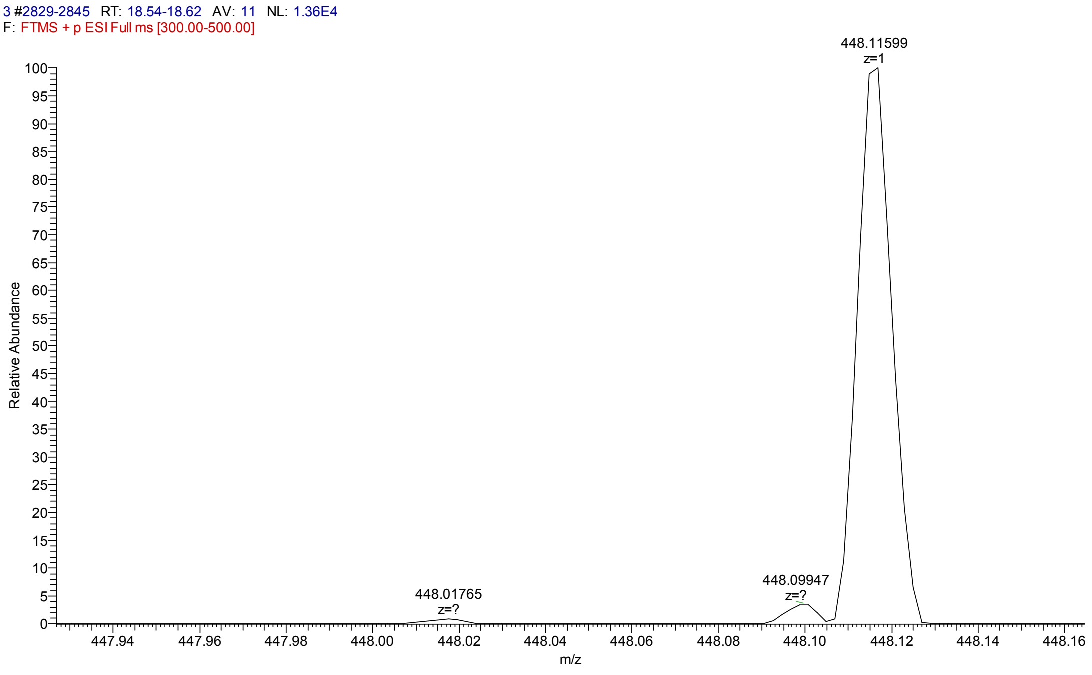

Figure S4. HPLC-MS detection of digestion of ODN-cbC.

(a) HPLC-MS extracted $[M+H]^+$ ion count for A, T, C, G, CB-C after digestion of DNA from the ODN-cbC. (b) HRMS (ESI+) of CB-C in HPLC-MS after digestion, HRMS $C_{19}H_{17}ClN_5O_4S^+$ $[M+H]^+$ calculated 446.06843, found 446.06617. (c) The isotopic distributions (M+2 isotopes) of the modified nucleoside was found 448.11599.

a

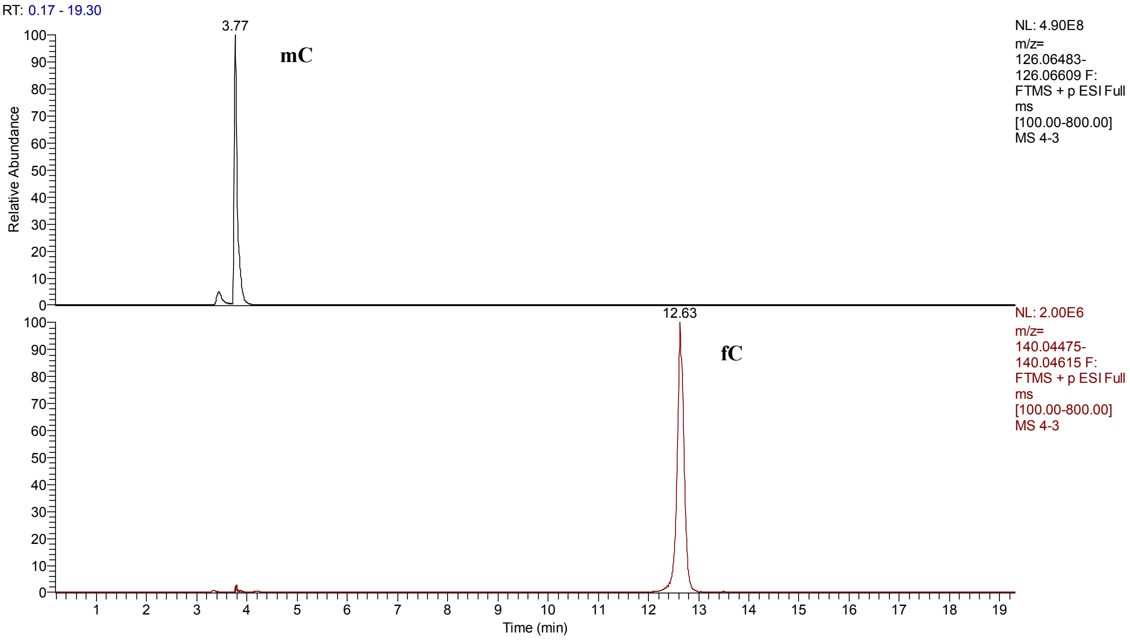

b

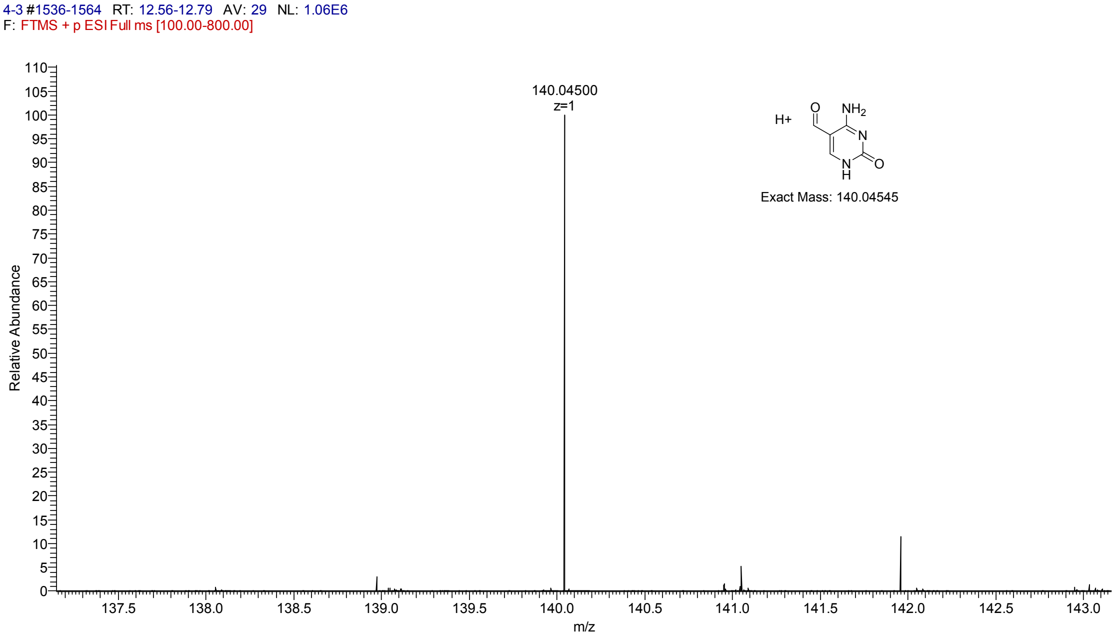

Figure S5. HPLC-MS detection of γ-irradiated 5mC solution.

(a) HPLC-MS extracted $[M+H]^+$ ion count for 5mC, 5fC in the γ-irradiated 5mC solution. (b) HRMS (ESI+) of 5fC in HPLC-MS, HRMS $C_5H_6N_3O_2^+$ $[M+H]^+$ calculated 140.04545, found 140.04500.

a

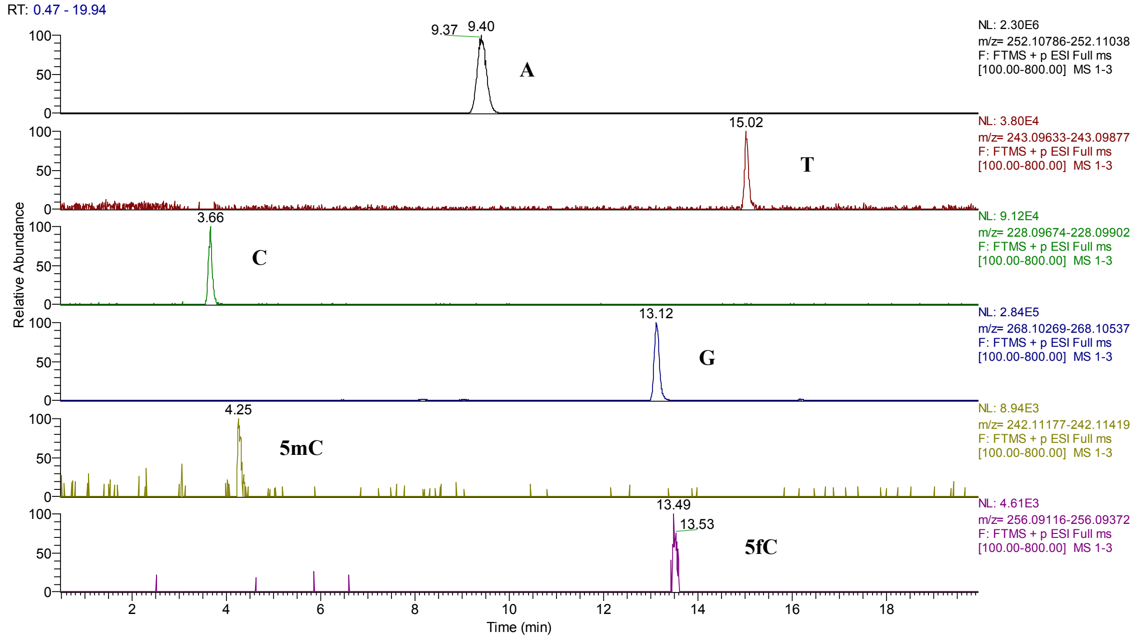
RT: 0.47 - 19.94
9.37
9.40
A
NL: 2.30E6
m/z= 252.10786-252.11038
F: FTMS + p ESI Full ms
[100.00-800.00] MS 1-3
15.02
T
NL: 3.80E4
m/z= 243.09633-243.09877
F: FTMS + p ESI Full ms
[100.00-800.00] MS 1-3
3.66
C
NL: 9.12E4
m/z= 228.09674-228.09902
F: FTMS + p ESI Full ms
[100.00-800.00] MS 1-3
13.12
G
NL: 2.84E5
m/z= 268.10269-268.10537
F: FTMS + p ESI Full ms
[100.00-800.00] MS 1-3
4.25
5mC
NL: 8.94E3
m/z= 242.11177-242.11419
F: FTMS + p ESI Full ms
[100.00-800.00] MS 1-3
13.49
13.53
5fC
NL: 4.61E3
m/z= 256.09116-256.09372
F: FTMS + p ESI Full ms
[100.00-800.00] MS 1-3
Relative Abundance
Time (min)

b

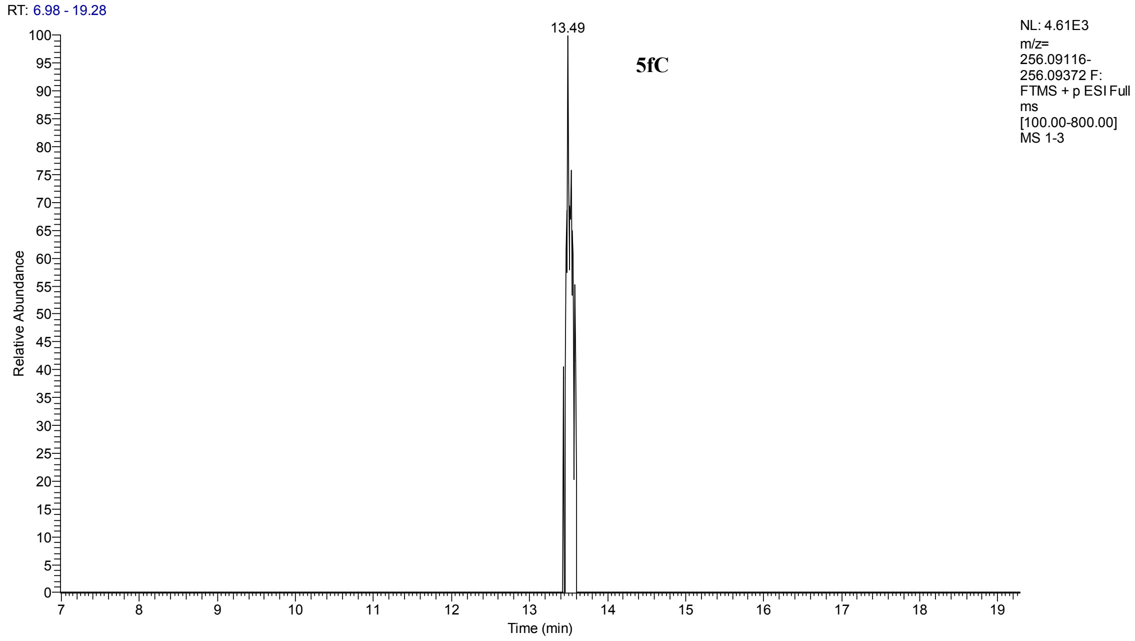
RT: 6.98 - 19.28
13.49
5fC
NL: 4.61E3
m/z=
256.09116-
256.09372 F:
FTMS + p ESI Full
ms
[100.00-800.00]
MS 1-3
Relative Abundance
Time (min)

c

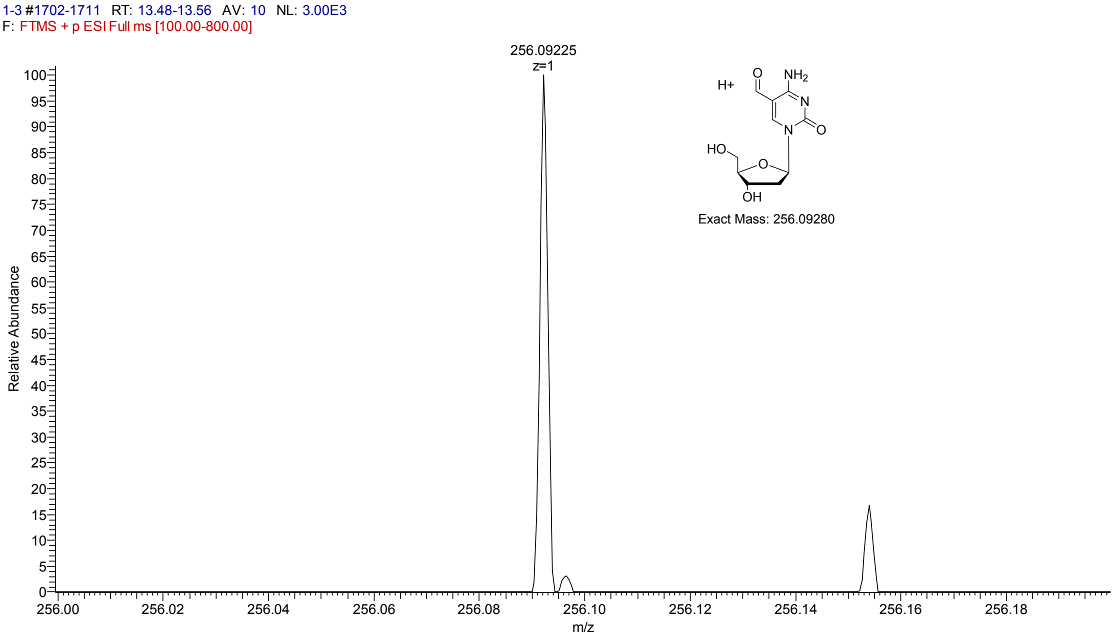

d

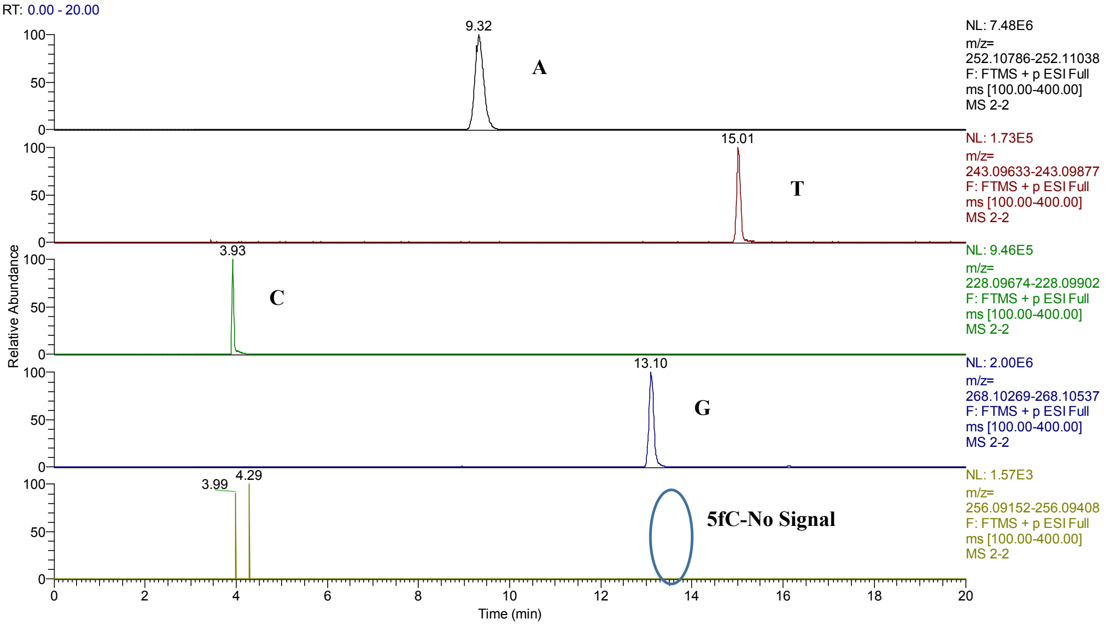

Figure S6. HPLC-MS detection of digestion of γ-irradiated ODN-5mC and its control solution. (a) HPLC-MS extracted $[M+H]^+$ ion count for A, T, C, G, 5mC and 5fC in the γ-irradiated ODN-5mC solution. (b) Zoom in details of 5fC signal in a. (c) HRMS (ESI+) of 5-formyl-2'-deoxycytidine in HPLC-MS, HRMS $C_{10}H_{14}N_3O_5^+$ $[M+H]^+$ calculated 256.09280, found 256.09225. (d) HPLC-MS extracted $[M+H]^+$ ion count for A, T, C, G and 5fC in the γ-irradiated ODN-C solution.

6. LC-MS/MS quantification

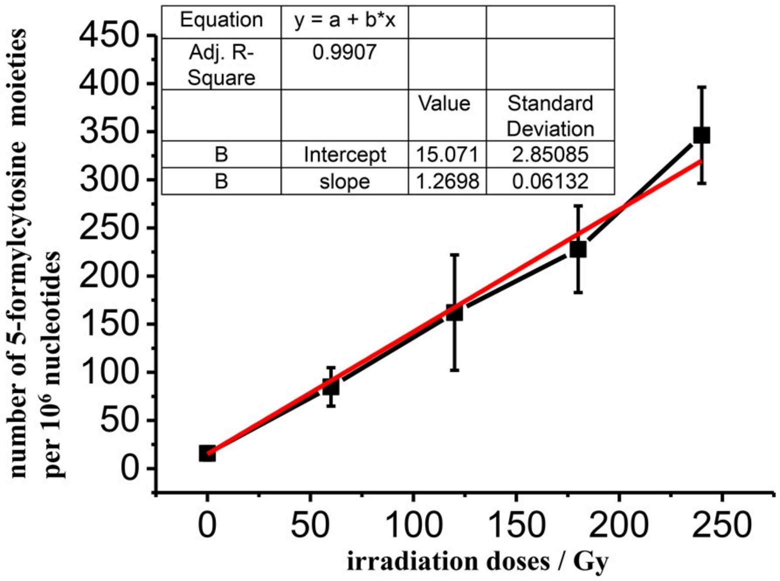

Figure S7. LC-MS/MS quantification of 5fC in γ-irradiated calf thymus DNA at different irradiation doses (0–240 Gy)[2].

7. UV absorption spectra and fluorescent emission spectra

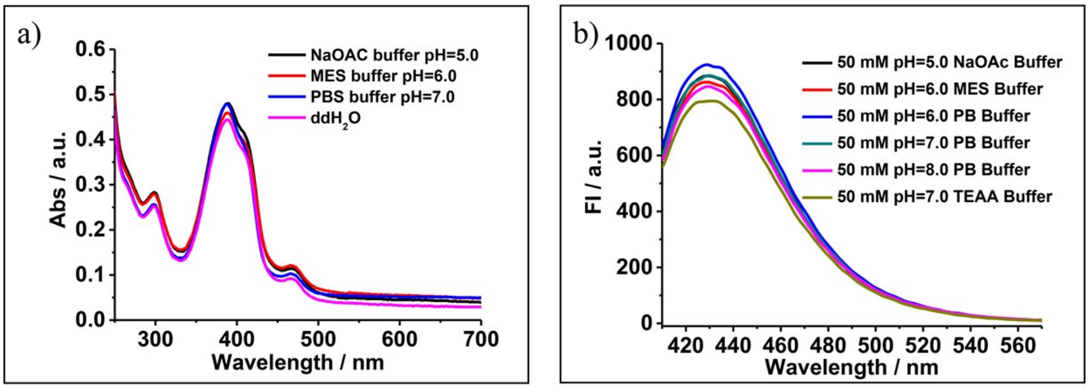

Figure S8. (a) UV absorption spectra of CB-C in the different buffer solutions; (b) Fluorescent emission spectra of CB-C in the different buffer solutions (λ_{ex}: 389 nm).

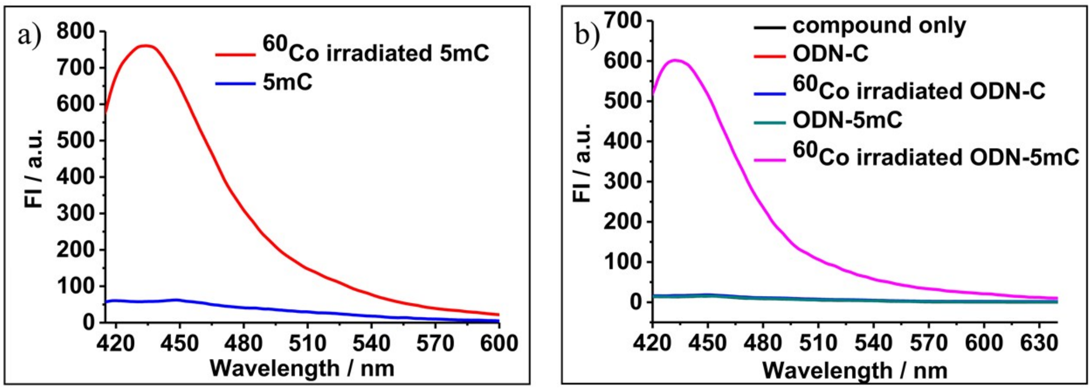

Figure S9. (a) Fluorescence emission spectra of γ-irradiated 5mC and 5mC after treatment with CBAN (λ_{ex}: 389 nm); (b) Fluorescent emission spectra of ODN-C, γ-irradiated ODN-C, ODN-5mC and γ-irradiated ODN-5mC after treatment with CBAN (λ_{ex}: 389 nm).

8. Sanger sequencing

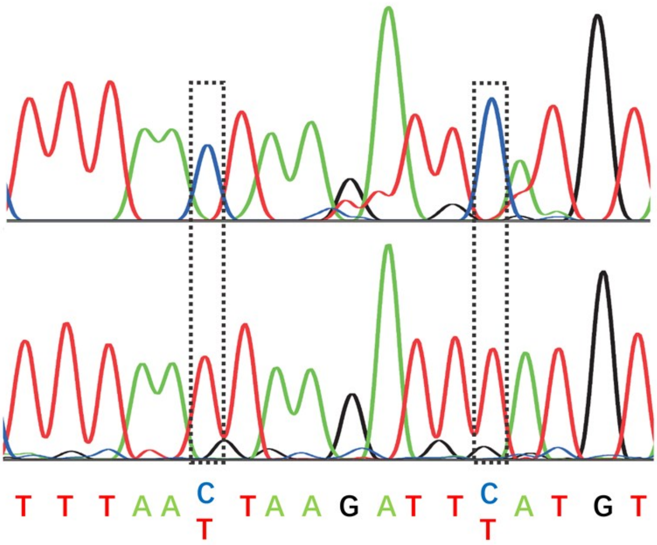

Figure S10. Sanger sequencing

Sanger sequencing analysis of before and after CBAN-treated 80-mer oligonucleotides containing two sites of 5fC. The original 5fC sites are surrounded by dotted lines.

9. Illumina sequencing

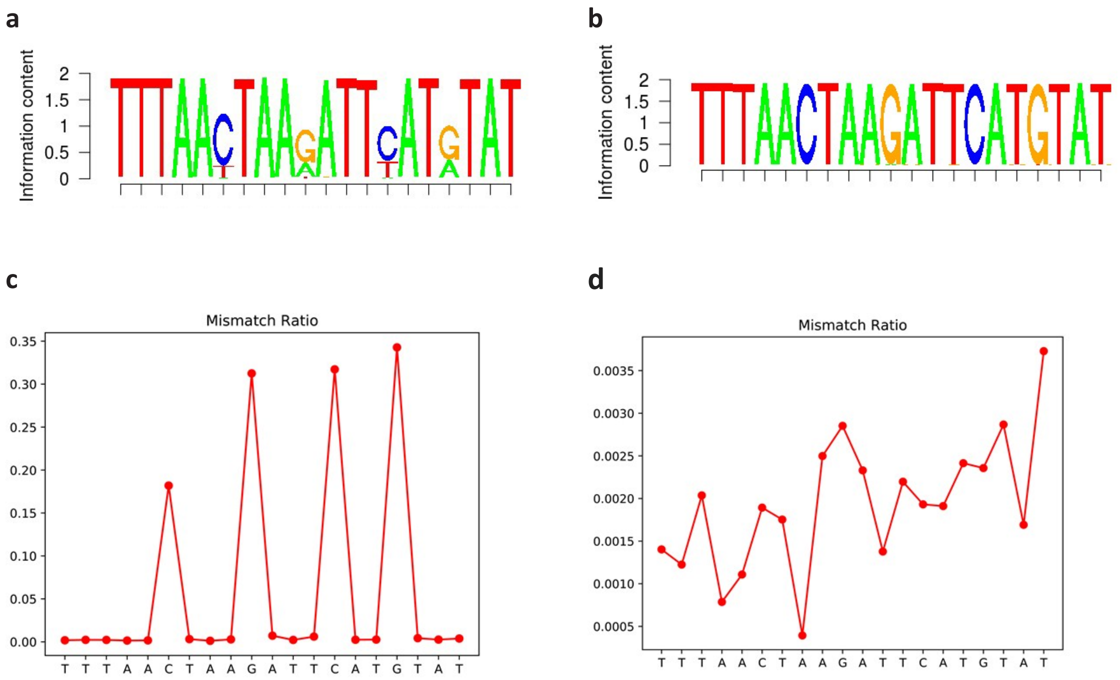

Figure S11. Illumina sequencing

Sequence logo of untreated (b) and treated with CBAN (a) showed the conversion ratio of four sites of 5fC; mismatch ratio of untreated (d) and treated with CBAN (c) showed the mismatch ratio of four sites of 5fC.

10. Synthesis

2-amino-4-chlorobenzenethiol (1.58 g, 10 mmol) and malononitrile (660 mg, 10 mmol) were dissolved in 10 mL ethanol in a 25 mL round bottom flask. Then, 1 mL acetic acid was added dropwise while the reaction mixture was kept stirring and stayed at room temperature. After 4 h, the precipitate was filtered off and collected through washed with cold ethanol and dried under vacuum to yield 1.38 g (66% yield) as a colorless solid. ^{1}H NMR (400 MHz, $CDCl_3$) δ = 8.04 (d, *J* = 1.9, 1H), 7.82 (d, *J* = 8.6, 1H), 7.44 (dd, *J* = 8.6, 2.0, 1H), 4.25 (s, 2H). ^{13}C NMR (100 MHz, $CDCl_3$) δ = 160.26, 153.65, 133.69, 132.92, 126.64, 123.32, 122.51, 114.69, 23.34. HRMS (ESI+) $C_9H_6ClN_2S^+$ $[M+H]^+$ calculated 208.99347, found 208.99403.

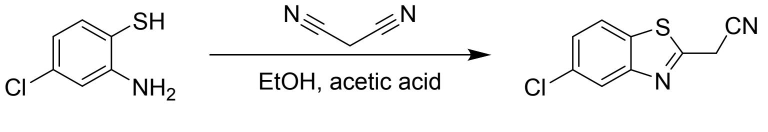

Figure S12. Synthesis of 2-(5-chlorobenzo[d]thiazol-2-yl)acetonitrile (CBAN).

2-(5-chlorobenzo[d]thiazol-2-yl)acetonitrile (41.6 mg, 0.20 mmol) and 5-formyl-2'-deoxycytidine[3] (51 mg, 0.20 mmol) were dissolved in 20 mL methanol which containing 0.1% piperidine. After 12 h stirring at 50°C, the precipitate was isolated by filtration and washed by cold methanol to yield 53 mg (60%) as a yellow powder. ^{1}H NMR (300 MHz, DMSO-*d6*) δ = 10.32 (s, 1 H), 8.18 – 8.03 (m, 4H), 8.00 (s, 1H), 7.44 (d, *J* = 8.6 Hz, 1 H), 6.03 (s, 1H), 5.92 (m, 1 H), 5.14 (m, 1H), 4.31 (s, 1H), 4.23 (m, 1H), 4.07 (d, *J* = 12.6 Hz, 1H), 3.89 (d, *J* = 11.9 Hz, 1H), 2.40 – 2.17 (m, 2H). ^{13}C NMR (100 MHz, DMSO-*d6*) δ = 169.32, 156.70, 154.22, 149.91, 149.54, 140.18, 131.56, 131.48, 125.34, 123.52, 121.67, 104.07, 102.37, 90.38, 88.10, 83.66, 71.89, 71.50, 46.29. HRMS (ESI+) $C_{19}H_{17}ClN_5O_4S^+$ $[M+H]^+$ calculated 446.06843, found 446.06756.

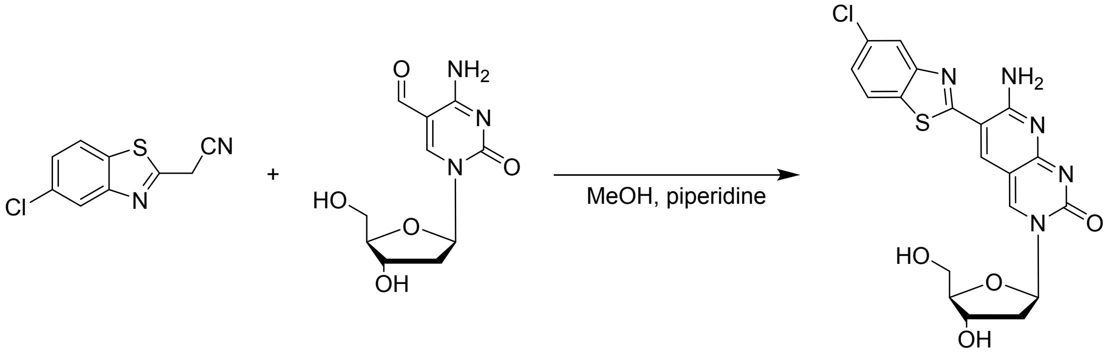

Figure S13. Synthesis of 5-formyl-2'-deoxycytidine-CBAN adduct (CB-C).

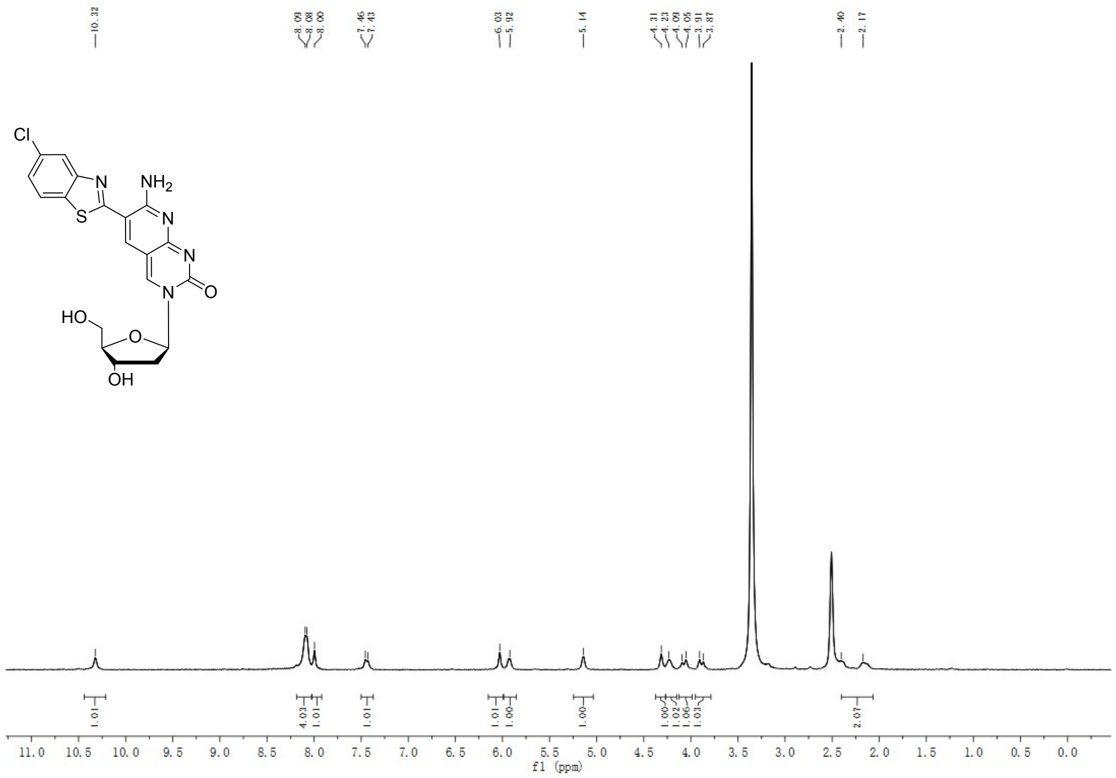

^{1}H NMR (in DMSO-*d6*)

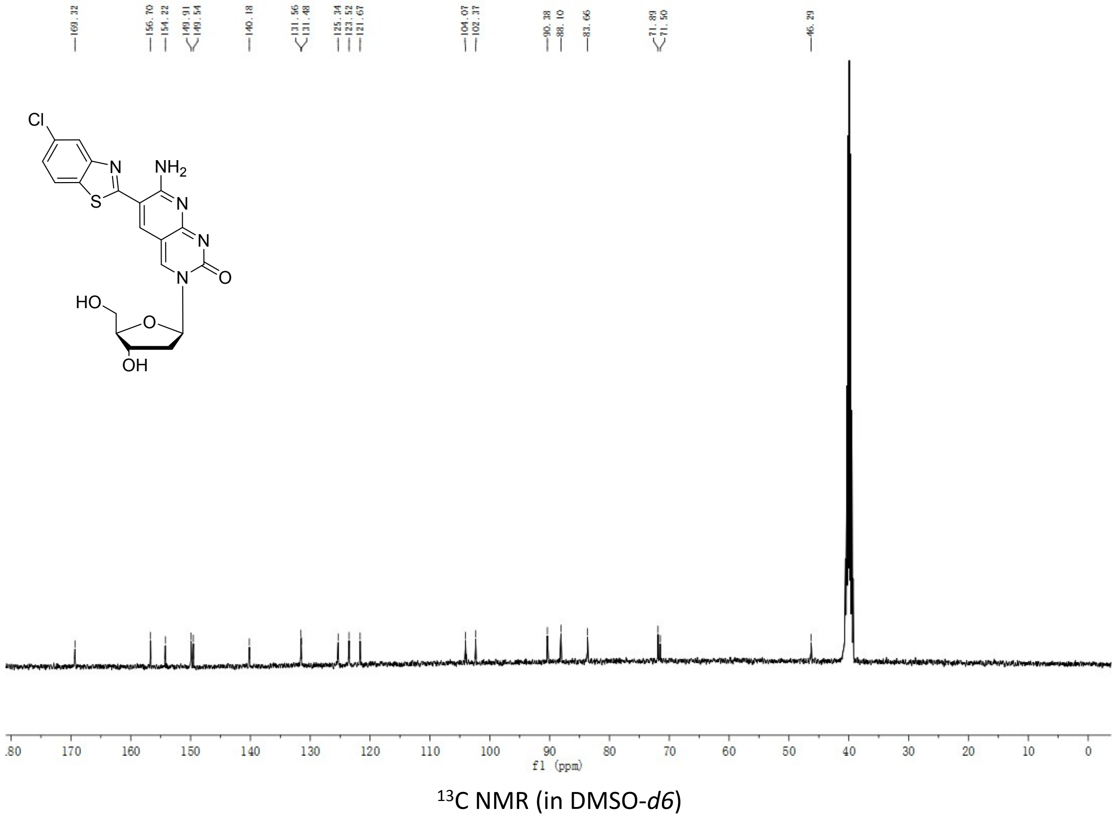

^{13}C NMR (in DMSO-*d6*)

2-(5-chlorobenzo[d]thiazol-2-yl)acetonitrile (25 mg, 0.12 mmol) and 5-formyl-2'-deoxyuridine[4] (31 mg, 0.12 mmol) were dissolved into 15 mL methanol which containing 0.1% piperidine. After 1 h stirring at 50°C, the precipitate was isolated by filtration and washed by cold methanol to yield 48 mg (90%) as a yellow powder. ^{1}H NMR (300 MHz, DMSO-*d6*) δ = 12.01 (s, 1H), 9.00 (s, 1H), 8.29 – 8.07 (m, 3H), 7.55 (dd, *J* = 8.5, 1.5 Hz, 1H), 6.16 (t, *J* = 6.5 Hz, 1H), 5.32 (d, *J* = 3.2 Hz, 1H), 4.97 (s, 1H), 4.28 (s, 1H), 3.92 (d, *J* = 2.1 Hz, 1H), 3.63 (s, 2H), 2.35 – 2.15 (m, 2H). ^{13}C NMR (100 MHz, DMSO-*d6*) δ = 165.63, 161.71, 154.25, 149.47, 143.49, 140.28, 133.26, 132.27, 126.52, 124.32, 122.74, 116.40, 107.18, 102.12, 88.77, 86.57, 71.11, 61.72, 40.85. HRMS (ESI+) $C_{19}H_{16}ClN_4O_5S^+$ $[M+H]^+$ calculated 447.05244, found 447.05125.

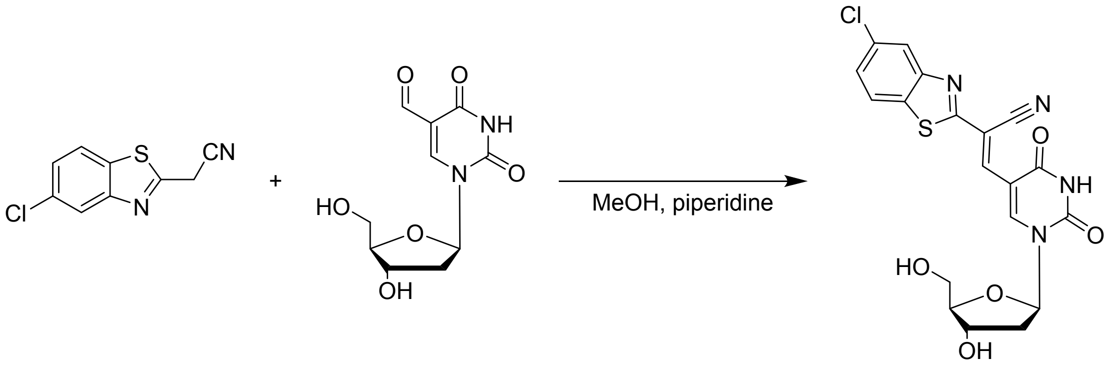

Figure S14. Synthesis of 5-formyl-2'-deoxyuridine-CBAN adduct (CB-U).

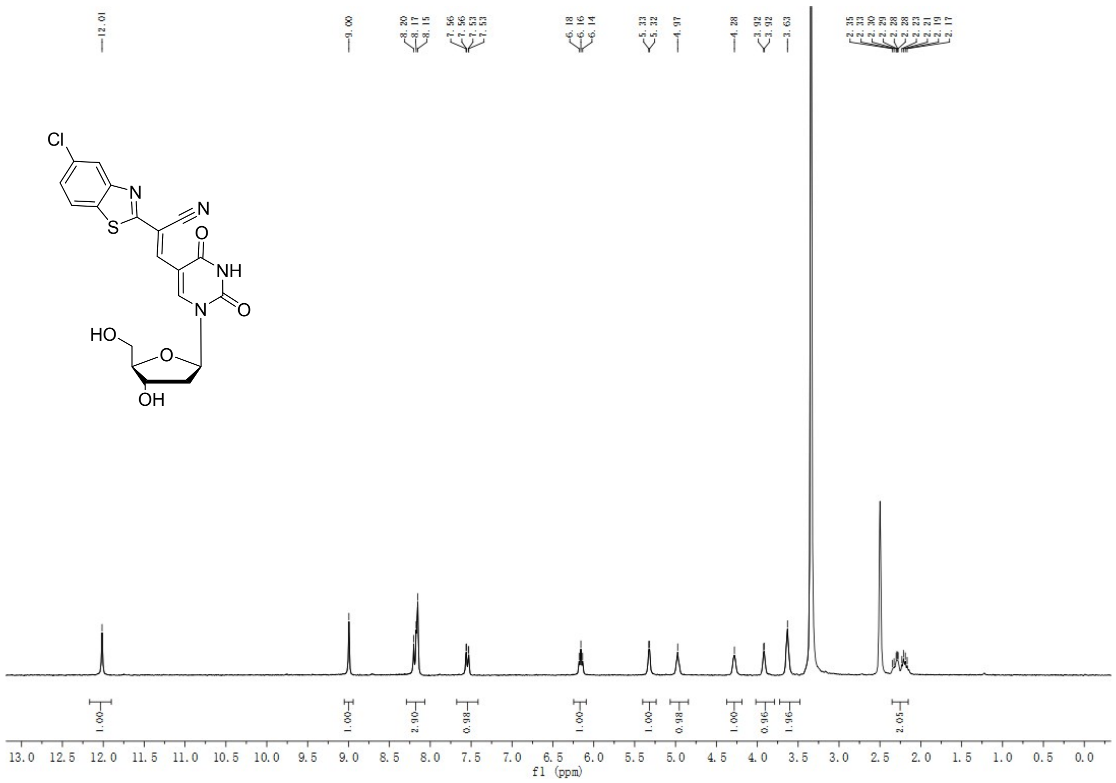

^{1}H NMR (in DMSO-*d6*)

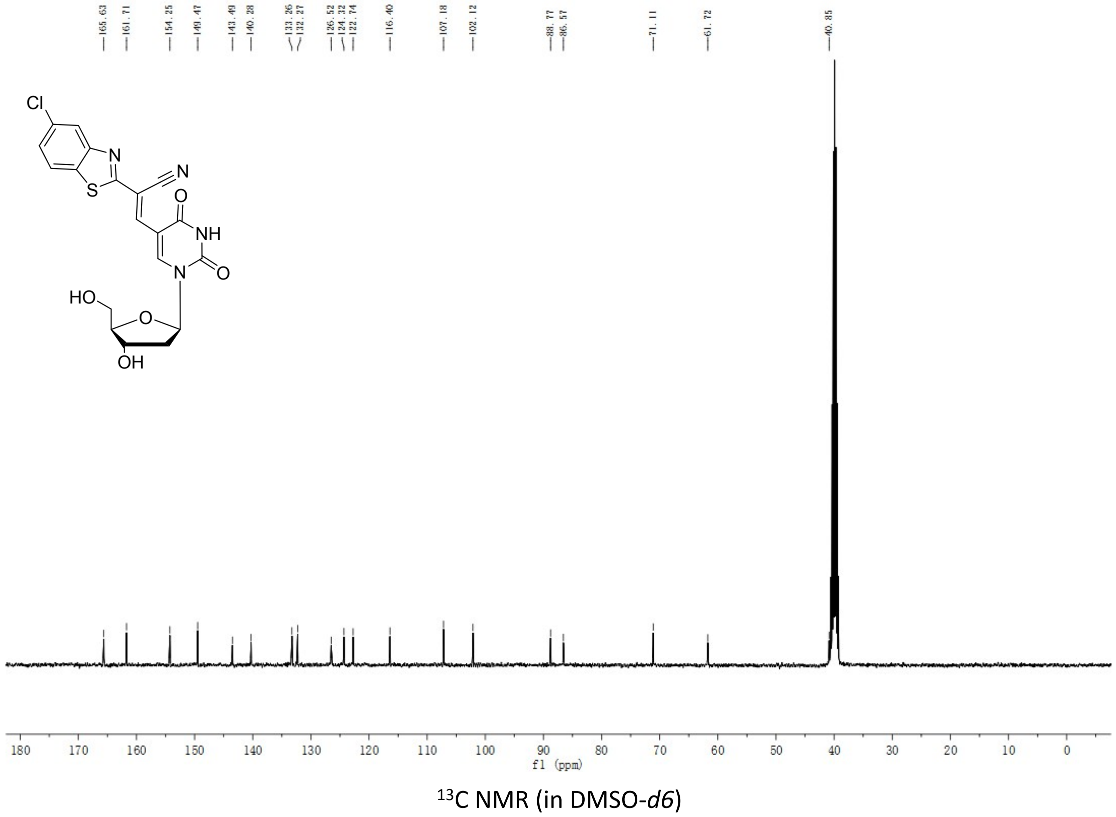

^{13}C NMR (in DMSO-*d6*)

11. Table of oligonucleotides sequences.

Oligomer	Sequence (from 5'to 3')
ODN-C	GACTCAA**C**AGCCGTA
ODN-AP	GACTCAA**AP**AGCCGTA
ODN-5fU	GACTCAA**5fU**AGCCGTA
ODN-5hmU	GACTCAA**5hmU**AGCCGTA
ODN-5mC	GACTCAA**5mC**AGCCGTA
ODN-5hmC	GACTCAA**5hmC**AGCCGTA
ODN-5fC	GACTCAA**5fC**AGCCGTA
ODN2-C	AAATCA**C**CCTATCCTCCTTCAGGACCAACGTAC
ODN2-5fC	AAATCA**5fC**CCTATCCTCCTTCAGGACCAACGTAC
80-SS-fU	TCCTCGGCGGTGTTGCTCTCTGTTGTGCCTCCGCCCG**5fU**CA<br>GGCAG**5fU**GGGCAGGACAAGGACGCAGAGCCACAGCCAAGAA
80-SS-T	TCCTCGGCGGTGTTGCTCTCTGTTGTGCCTCCGCCCG**T**CA<br>GGCAG**T**GGGCAGGACAAGGACGCAGAGCCACAGCCAAGAA
80-SS-fC	CCTATCATCTTATATCTACTACTACTACCTTTAA**5fC**TAAGA<br>TT**5fC**ATGTATAGAATAGATTTAGAGGATTTAGTAGATTTAG
80-SS-C	CCTATCATCTTATATCTACTACTACTACCTTTAA**C**TAAGA<br>TT**C**ATGTATAGAATAGATTTAGAGGATTTAGTAGATTTAG
80bp ds ODN-fU	a) TCCTCGGCGGTGTTGCTCTCTGTTGTGCCTCCGCCCG**5fU**CAGG<br>CAG**5fU**GGGCAGGACAAGGACGCAGAGCCACAGCCAAGAA<br>b) TTCTTGGCTGTGGCTCTGCGTCCTTGTCCTGCCCAC**5fU**GCC**5fU**<br>GACGGGCGGAGGCACAACAGAGAGCAACACCGCCGAGGA
80bp ds ODN-fC	a) CCTATCATCTTATATCTACTACTACTACCTTTAA**5fC**TAAGA<br>TT**5fC**ATGTATAGAATAGATTTAGAGGATTTAGTAGATTTAG<br>b) CTAAATCTACTAAATCCTCTAAATCTATTCTATA**5fC**ATGAAT**5fC**<br>TTAGTTAAAGGTAGTAGTAGTAGATATAAGATGATAGG
80bp ds ODN-C	a) GCTCGCTTTGTTGGTTTCCTTGTTCTCTGTGCCCACTGCCTG<br>ACGGGCGGAAAGCAGCGCGAGCAAGCGAGACAGGACAC<br>b) GTGTCCTGTCTCGCTTGCTCGCGCTGCTTTCCGCCCGTCAG<br>GCAGTGGGCACAGAGAACAAGG AAACCAACAAAGCGAGC
ODN2- Primer	5'-FAM-CGTTGGTCCTGAAGGAGGATAGG

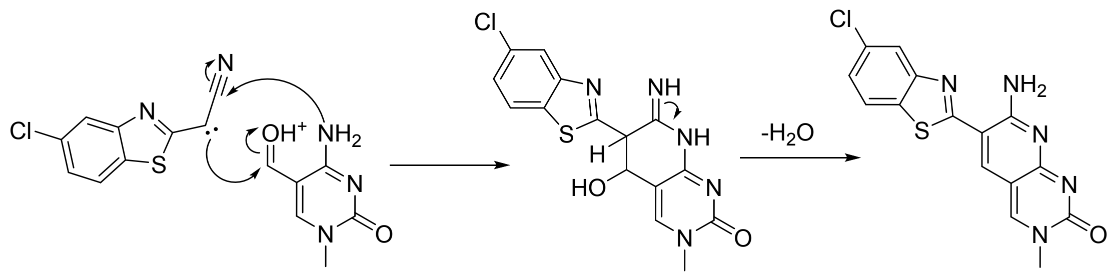

Scheme S1. The perhaps reaction mechanism between CBAN and 5fC in DNA.

12. References

1. Liu, C.; Chen, Y.; Wang, Y.; Wu, F.; Zhang, X.; Yang, W.; Wang, J.; Chen, Y.; He, Z.; Zou, G.; Wang, S.; Zhou, X. *Nano Res.* 2017, **10**, 2449-2458.
2. Tang, Y.; Xiong, J.; Jiang, H.; Zheng, S.; Feng, Y.; Yuan, B. *Anal. Chem.* 2014, **86**, 7764.
3. Hu, J.; Xing, X.; Xu, X.; Wu, F.; Guo, P.; Yan, S.; Xu, Z.; Xu, J.; Weng, X.; Zhou, X. *Chem. Eur.J*. 2013, **19**, 5836.
4. Guo, P.; Xu, X.; Qiu, X.; Zhou, Y.; Yan, S.; Wang, C.; Lu, C.; Ma, W.; Weng, X.; Zhang, X.; Zhou, X. *Org. Biomol. Chem.* 2013, **11**, 1610.
